# Type D personality as a predictor of poor health outcomes in patients with cardiovascular disease

**DOI:** 10.1007/s12471-017-0949-4

**Published:** 2017-01-24

**Authors:** E. M. Pluijmers, J. Denollet

**Affiliations:** Center of Research on Psychological and Somatic Disorders (CoRPS), Tilburg University, Tilburg, The Netherlands

To the Editor,

With great interest we have read the study by Al-Qezweny et al. [1]. In this prospective study the association between Type D personality and depression was investigated in patients who have been treated with percutaneous coronary intervention (PCI). The researchers found an increased risk for depression and anxiety in PCI patients with a Type D personality compared with PCI patients with a non-Type D personality.

This interesting study sheds more light on the complex relationship between depression and Type D personality. Type D personality is a multifaceted construct which consists of two components: negative affectivity and social inhibition [2]. Negative affectivity is composed of three facets: dysphoria, anxious apprehension, and irritability [2]. In other words, dysphoria is one of the constituting facets of negative affectivity, and the association between Type D personality and depression also validates the negative affectivity component of Type D. This partial overlap was found in several studies which, however, also supported the psychometric distinctiveness of the Type D personality as compared with depression [2, 3]. Overall, research indicates that Type D personality is a predictor or vulnerability factor of depression [3], which is also supported by the findings reported by Al-Qezweny et al.

As stressed above, Type D personality not only focuses on negative affectivity but also on social inhibition. To explore the relationship between social inhibition and depression, previous research examined four personality groups based on high and low scores of social inhibition and negative affectivity in relation to depression [3]. Depressive disorder and depressive symptoms were more prevalent in the Type D group characterised by high scores for both negative affectivity and social inhibition, as compared with patients who scored high on negative affectivity but low on social inhibition. Hence, the combination of social inhibition and negative affectivity within the Type D personality construct further increased the risk of depression, above and beyond the main effect of the negative affectivity component by itself [3].

Other empirical evidence showed that Type D personality was also an independent predictor of major adverse cardiac events in patients with coronary artery disease, even after adjustment for depression [2]. This association could be explained by dysregulations of the neuroendocrine, autonomic nervous, and immune systems as was found in several studies. For example, Type D personality has been associated with heightened levels of disease-promoting biomarkers (e. g. oxidative stress and macrophage activity) while controlling for depression [2, 3]. This indicates Type D and depression are only partly overlapping constructs, that may independently predict cardiac events.

The results of the study of Al-Qezweny et al. [1] provide important new evidence for the potential value of Type D personality as a predictor of poor health outcomes in patients with cardiovascular disease.
